# Midcareer Medical School Research Faculty Perspectives on Vitality and Professionalism During the COVID-19 Pandemic

**DOI:** 10.1001/jamanetworkopen.2021.20642

**Published:** 2021-08-13

**Authors:** Linda H. Pololi, Vasilia Vasiliou, Kimberly Bloom-Feshbach

**Affiliations:** 1 National Initiative on Gender, Culture and Leadership in Medicine: C-Change, Brandeis University, Waltham, Massachusetts; 2 School of Management, Clark University, Worcester, Massachusetts; 3 Medicine, Section of Hospital Medicine, Division of General Internal Medicine, Weill Cornell Medicine, New York, New York

## Abstract

**Importance:**

As medical faculty have central roles during the COVID-19 pandemic, it is important to study the pandemic’s association with the vitality and careers of medical school faculty.

**Objective:**

To examine how the COVID-19 pandemic affected midcareer research faculty in academic medicine.

**Design, Setting, and Participants:**

This qualitative study included medical school faculty who participated in the C-Change Mentoring and Leadership Institute. All US medical school faculty recipients of recent National Institutes of Health (NIH) RO1, RO1-equivalent, and K awards were invited to apply to the institute. The 99 applicants who met inclusion criteria were stratified by degree (MD or MD/PhD vs PhD), gender, and race/ethnicity. Enrollment was offered to applicants randomly selected for 40 spots, demographically balanced by sex, underrepresented in medicine minority (URMM) status, and degree. In April 2020, an inquiry was emailed to faculty enrolled in the institute requesting responses to questions about meaning in work, career choice, and values. A qualitative analysis of narrative data responses, using grounded theory, was undertaken to determine key themes. This study is part of a NIH-funded randomized trial to test the efficacy of a group peer mentoring course for midcareer faculty and study the course’s mechanisms of action.

**Main Outcomes and Measures:**

Key themes in data.

**Results:**

Of 40 enrolled participants, 39 responded to the inquiry, for a response rate of 97%. The analytic sample included 39 faculty members; 19 (47%) were women, 20 (53%) identified as URMM, and 20 (53%) had an MD or MD with PhD vs 19 (47%) with PhD degrees. Key themes in the data that emerged describing faculty lived experience of the pandemic included increased meaningfulness of work; professionalism and moral responsibility; enhanced relationships with colleagues; reassertion of career choice; disrupted research; impact on clinical work; attention to health disparities, social justice and advocacy; increased family responsibilities; psychological stress; and focus on leadership.

**Conclusions and Relevance:**

During the pandemic, diverse PhD and physician investigators reported increased meaningfulness in work and professionalism and enhanced relationships, all intrinsic motivators associated with vitality. Working during the pandemic appears to have produced intrinsic rewards positively associated with vitality, in addition to adverse mental health effects. These findings have implications for combatting burnout and retaining investigators in the future.

## Key Points

**Question:**

How do midcareer medical school faculty perceive the impact of the COVID-19 pandemic on their lives and their work?

**Findings:**

In this qualitative study of 39 midcareer research faculty, dominant themes included finding increased meaningfulness of work; a sense of professionalism and moral responsibility; enhanced relationships with colleagues; reassertion of career choice; disrupted research; impact on clinical work; attention to health disparities, social justice, and advocacy; increased family responsibilities; psychological stress; and focus on leadership.

**Meaning:**

In this study of a diverse group of midcareer medical school faculty, the experience of working during the pandemic appeared to have had important positive impacts on physician investigators and PhD scientists, contributing to their vitality and professional dedication that were associated with intrinsic motivators.

## Teaser

This qualitative study examines how the COVID-19 pandemic affected midcareer research faculty in academic medicine.

## Introduction

Faculty with high vitality are essential to the missions of academic health centers. Studies of faculty vitality and the existing culture of academic medicine document the nonrelational, very competitive, and emotionally and intellectually demanding workplace.^1,2,3,4,5,6,7,8^ Additionally, among faculty, many women and members of racial and ethnic groups underrepresented in medicine report the added burden of gender^9,10,11,12,13,14^ and racial discrimination.^15,16,17,18,19^ As COVID-19 began to overwhelm hospitals, we recognized that the pandemic might powerfully alter the lives and work of academic medical faculty. The purpose of this study was to understand how research faculty perceived the impact of the COVID-19 pandemic.

One month after the World Health Organization declared COVID-19 a pandemic in March 2020, we collected data from faculty who had enrolled in a career development course.^20^ Our course is part of a research project funded by the National Institutes of Health (NIH) seeking to contribute to the science of mentoring and increase the diversity of the national research workforce.

## Methods

Using qualitative research methods and following the Standards for Reporting Qualitative Research (SRQR) reporting guideline,^21^ we sought to understand the lived experience of faculty and how they perceived the effect of the pandemic on their lives and work, as it occurred, rather than in an experimental situation.^22,23^ This study was approved by the Brandeis University institutional review board, and written informed consent was provided by all participants, including consent for collection of qualitative narrative data.

### Sampling Strategy

The sample consisted of 40 midcareer medical school faculty who had enrolled in the Brandeis University–based C-Change Mentoring and Leadership Institute, a yearlong career development course scheduled to meet quarterly for sessions lasting 2 to 3 days each. One cohort of 20 faculty started the program in December 2020, and the second cohort enrolled for December 2021.

Using the NIH RePorter database, we invited all US medical school faculty recipients of a recent R01, R01-equivalent, or K award to apply. Among the applicants, we constructed an eligible list of 99 midcareer faculty who met the inclusion criteria, including demonstrated substantial research aspirations by having been awarded their first NIH R01 or R01-equivalent grant or similarly competitive grant in the prior 3 years or an NIH K award in the prior 4 years. Using a stratified (by degree, gender, and race/ethnicity) randomized selection approach, we offered eligible applicants a place in the institute (acceptance rate, 87%). Race/ethnicity categories were NIH-defined and were self-reported. We divided race/ethnicity categories into the underrepresented in medicine minority (URMM) group (American Indian or Alaska Native, Black or African American, Latinx/Hispanic, and Native Hawaiian or other Pacific Islander) and the well-represented in medicine group (Asian and White, non-Hispanic). Enrollees were randomly assigned to 1 of 2 cohorts. Each cohort was demographically stratified and balanced by doctoral degree (MD or MD/PhD vs PhD). Half were women and half were URMM group members. The physician investigators and PhDs represented 16 US states.

### Data Collection and Analysis

In early April 2020 (8 months prior to the course beginning), the primary investigator sent an email to the 40 enrolled faculty inviting them to respond to three questions: (1) how has the coronavirus affected the meaning you find in your work? (2) how are you feeling about your role and career choice now with the COVID-19 crisis? and (3) how are your values being impacted/tested in these times? Most faculty answered promptly via email. Two reminders were sent.

Narrative responses to the questions were deidentified and aggregated. To minimize bias, 3 coders (L.P., V.V., K.B.F.) with different disciplinary backgrounds intentionally set aside their own experiences with the COVID-19 pandemic^24^ and developed codes by repeatedly reading the aggregated data to develop understanding and meaning without reference to degree or demographic variables. The coders developed a final set of 47 codes by consensus to ensure intercoder consistency.^25^ We stored data using Excel version 16.50 (Microsoft Corp). Analysis involved data reduction and condensation, from which we identified themes and patterns emergent in the coded data. We used an inductive process, in line with grounded theory.^26^ To verify our conclusions, 2 investigators (L.P. and K.B.F.) returned to the aggregated data, reevaluating the findings to develop intersubjective consensus and to assess for thematic variation in demographic groups of respondents.

## Results

Of the 40 faculty enrollees, 39 wrote narrative responses (response rate, 97%). Table 1 shows demographic attributes of respondents. Overall, 19 respondents (47%) were women, 20 (53%) identified as URMM, and 20 (53%) had an MD or MD with PhD vs 19 (47%) with PhD degrees. We describe themes that emerged from the data. All themes represented the perspectives of PhD and physician scientists, both male and female. Quotations in the text were selected as illustrative of the analytic findings. Table 2 and Table 3 present additional quotes representing the perspectives of multiple respondents.

**Table 1.** Demographic Attributes of Midcareer Medical School Faculty Respondents

Gender and race/ethnicity	Respondents by degree, No. (%) (N = 39)	
	MD or MD/PhD	PhD
**Male**		
URMM	5 (13)	5 (13)
WRM	5 (13)	5 (13)
**Female**		
URMM	5 (13)	5 (13)
WRM	5 (13)	4 (10)

Abbreviations: URMM, underrepresented in medicine minority; WRM, well-represented in medicine.

**Table 2.** Quotations Written by Midcareer Medical School Faculty and Stratified by Theme

Theme^a^	Quotation
Increased meaningfulness of work	“While my research is not in virology and infectious diseases, the current crisis emphasizes what we are doing every day of our life, which is battling a disease and trying to save lives or improve lives. While each of us are fighting our own battle, it is the first time that I experience the scientific community mobilizing for an all-out war against COVID-19.”
	“Yes, the coronavirus has affected the meaning I find in my work in that the nature of my work makes me understand the basic biology of the coronavirus. It also shows me that while many people do not appreciate the importance of basic research in providing drugs and treatments that help us live longer healthier lives, basic research is nonetheless very important and rewarding.”
	“I have always tried to focus my work on saving lives, but this goal is currently much more immediate. This goal feels very meaningful to me!”
	“I have found my work to be even more meaningful than before.”
	“As a physician scientist, I do appreciate more the role of science in everyday life and now have the conviction to defend it.”
	“I have always found both my clinical and research work to be quite meaningful. Therefore, I have not found coronavirus to have any change in my sense of meaningfulness.”
	“It makes me appreciate the small things in life such as food and roof over my head, and being grateful for all that we have, our families, friends, coworkers and our health. I pray for my clinical colleagues on the front lines every day.”
Professionalism	“So when I wake up every morning I tell myself I need to go to work because there are a lot of people, patients, and physicians, that depend on my work and that need me.”
	“I am hoping that our lab[oratory] can make some positive impact in these dark times.”
	“While it is a stressful, risky position to be within the frontlines of diagnosing and managing patients with suspected COVID-19, it is also very rewarding to realize how relevant our work is. Being in the center of all and being able to contribute positively makes our sacrifice worthwhile.”
	“Most of my colleagues, and most of my institution’s leaders, are stepping up beautifully during this time. On the other hand, I have a few colleagues who are refusing to pitch in, presumably out of fears regarding safety, and I have found this incredibly challenging, both in terms of trying to withhold judgement and understand that everyone’s circumstances are different, and in terms of how I will return to ‘usual’ work knowing that some members of my team do not prioritize values and needs in the same way that I do.”
	“I feel honored to have the training that allows me to help in a situation such as this.”
	“I’m humble at the responsibility I have to participate in the delivery of health care.”
	“Overwhelmingly, I am glad to be in a role to be able to help fight this crisis. And I am proud to see physicians stepping up and reminding the world that we are here to help and that medicine is still a noble profession. I am confident that I am in the right profession. At the same time, I am envious of others who can stay home and snuggle with their families. And I am fearful that I or my loved ones might die.”
	“I am glad I am not an MD. [V]ery scared about the idea of being deployed—as some of my close colleagues have been.”
	“That is what is driving me in these difficult times—the opportunity to be a positive force.”
	“There is a major concern for this situation resulting in a greater gender divide in our careers. The majority of my female colleagues are really struggling to simply stay afloat (conversely, male colleagues complain of boredom and are submitting many papers and grants).”
Enhanced relationships with colleagues	“This has provided the opportunity for enhanced meaning in interactions with colleagues and particular trainees and mentees, as we all face this uncertainty together. Our medical community has also come together with enhanced wellness initiatives to help us share fears, guilt, joy, and victories with each other, and this has brought us closer as colleagues and as an overall community.”
	“I think the crisis has definitely brought our clinical and administrative units closer together, and we are more appreciative of the work that each one of us do. Feels like we are family.”
	“I am glad to be … part of a vibrant and passionate community at my institution that has responded as one voice but in many creative and complementary ways.”
Reassertion of career choice	“I am feeling that my role in public health is exactly where I want to be. [Al]though I am not directly involved in communities as a health service worker, my role as a behavioral research scientist is also integral to this crisis.”
	“The novel coronavirus pandemic has reiterated and reinforced why I chose this field.”
	“The pandemic has reasserted my commitment to my career in data science and clinical informatics. Data has been key in allowing us to respond to this crisis, but there is so much more that we could do.”
	“I am not on the frontline, but do my part coordinating from home … I did not need this crisis to appreciate my career choice, but I am glad to be able to understand what is going on in much details.”
	“This had made me rethink whether I want to get back on the hamster wheel of academia when this public health crisis is over. My work is important, but I ain’t crazy about the working conditions. (I’m on soft money.)”

^a^ Each theme includes representation of PhD and physician scientists, both male and female.

**Table 3.** Quotations Written by Midcareer Medical School Faculty Stratified by Theme

Theme^a^	Quotation
Impact on research	“We shut down the lab[oratory] 3 weeks ago and moved all of our work to off bench. While this time has been productive with regard to manuscript and grant writing, it is very crippling as we can’t continue to actively work to answering scientific questions we devoted our last years to.”
	“I’m feeling a bit depressed and disconnected. This is from having to close down the lab[oratory].”
	“[W]e have had to pivot some of our research areas to COVID-related questions, eg, how it impacts cancer patient care … [F]inding the flexibility in my career to be very helpful in this situation.”
	“It has made it very difficult to continue with my research, when so much of it does not seem to be as meaningful at the moment. Objectively, I know that it will become more meaningful than ever ... but in the moment, the clinical pull is greater, as I feel the need to do my part to care and prepare for surge of COVID19 patients.”
Impact on clinical work	“During the process of triaging essential and nonessential patients, I quickly realized that … 1. Many of my patient visits were deemed nonessential. 2. The ones that did need to be seen, could be handled by using telehealth (at least for this time period). My friends and I joke that our subspecialty is really ‘not essential’ apparently.”
	“In the moment, the clinical pull is greater, as I feel the need to do my part to care and prepare for surge of COVID-19 patients.”
	“It has helped to refocus on the most critical issues facing our patients.”
	“I value patients being able to heal, in part, through in-person interactions with their loved ones and friends. Isolation of inpatients from their families had been a difficult but necessary step to accept.”
	“I haven’t been called to help in the adult ICUs yet and I feel both excited and a little terrified of that prospect.”
Health disparities, social justice, and advocacy	“I am deeply committed to understanding the ways in which underserved communities are impacted and/or do not have a fair/just opportunity to achieve wellness.”
	“I have also been challenged to think of ways to help underserved communities, in the area and nationally, which are disproportionately being impacted (both health-wise and economically), during these hard times.”
	“I am reminded that being in spaces and circles where women are valued and uplifted is a core value.”
	“I hope that if I did see a glaring injustice I would speak up. (I have when it came to my pregnant fellow engaging in unnecessary direct patient care).”
	“It has been a great opportunity to help [another] country’s response to the crisis. I have had opportunity to advise the government on various issues related to control of the virus.”
	“The disparities I am witnessing secondary to COVID are quite similar to the disparities I witness in sub-Saharan Africa. They motivate me to find solutions to minimize disparities.”
	“The findings of disproportionate effects segregating along racial lines is not surprising but really very sad. Certainly provides ample opportunity to work toward closing those gaps.”
Family responsibilities	“I am a solo parent without other family members on site, it has been very difficult to keep up my productivity at home.”
	“I have to balance my desire to work in the frontlines with the need to protect an elderly parent and an immunocompromised spouse at home.”
	“This crisis has forced me to think a lot about the values we have as a family, what I would want for my kids if something happened to me (I made my Last Testament & Will at the beginning of this), the fact that I'm far away from my parents and what I would do if something happened to them.”
	“Even after a 12-hour shift, I still spend my last hour of the day before going to bed with my wife and children, who are on lockdown at home.”
	“I worry about so many things—the impact on my children, they are missing out on so many aspects of school, sport, social emotional development.”
Psychological stress	“It feels as though COVID is the only disease that matters in the world, and that is stressful. The non-COVID related work has ground to a halt.”
	“Forces me to prioritize personal well-being of staff and coworkers, and consider importance of a good team during periods of crises and change.”
	“At this stage, I am feeling very low (during these testing times) but I am determined to hang in there, do the best I can and come out stronger and help more young scientists in the future.”
	“Although there is less work, I am finding that the clinical work I am involved in can be more stressful. Also, my colleagues are equally stressed which makes communication more challenging.”
	“It has affected me tremendously. Before I left my previous position, I could not do a proper goodbye to patients in person. It was all done over the phone which really affected my mental health.”
Leadership	“I am feeling grateful for working at an organization that has had clear, transparent leadership, in a state with good governance. This has made the clinical impact manageable, but still difficult.”

Abbreviation: ICU, intensive care unit.

^a^ Each theme includes representation of PhD and physician scientists, both male and female.

### Increased Meaningfulness of Work

Finding work more meaningful was widely expressed by both physician investigators and PhDs working in different fields and disciplines, whether on the clinical frontlines with direct patient care or in the data sciences, research laboratories, clinical informatics, pathology, or global health. The respondents expressed excitement and enthusiasm associated with this enhanced clarity about the meaningfulness of their work. Faculty linked meaningfulness to the value of science, patient care, the essential nature of medical education and mentoring, and the realization of the contribution they were able themselves to make (Table 2).

- “These are extremely difficult times but also very exciting because the coronavirus has defined better than ever the meaning of my work.”
- “COVID-19 has significantly affected the meaning I find in my work. It made me realize that research I perform, my research team, my institute, as well as daily interaction with scientific community are the essential components of my life.”

Many respondents stated how COVID-19 had brought into focus the importance of the role of science in daily life, highlighting the significance of medical science, basic science, and SARS-CoV-2 science. The pandemic increased their determination to continue pursuing scientific goals.

- “It feels good to be at the scientific center of things and to know that one’s prior and future work is likely to be contributing to greater good.”
- “[P]hysicians—and in particular an academic physician scientist such as myself—are in the center of society's response to the pandemic, coordinating and informing mitigation efforts, driving research on diagnosis, prognostication, and therapies, and helping sick patients cope while improving their odds of survival. In every aspect of this global threat, medicine is at the forefront of our response. Again, this makes our work even more impactful and meaningful.”

Respondents described a number of other internally derived satisfying benefits related to COVID-19. These intrinsic factors included noticing and naming their own values and contributions, aligning their work with their own values, and gratitude.

- “Current events largely reaffirm my values in social equity (universal healthcare) and responsibility (environmental protection).”

### Professionalism

Another dominant theme in the data was the sense of professionalism and moral responsibility felt by respondents. Some respondents wrote about a rediscovery of their mission and altruism and how this affirmed their professional dedication, while others wrote about prioritizing their professional responsibilities even when incurring risk for their own family (Table 2).

- “Many people would think that being in New York City right now is hell, but I think it has been a blessing because I feel I am helping patients, and although many have died, many have also survived, and I will like to think I have contributed to save lives locally at my hospital, but also by publishing our observations we can help others that have not yet being affected as bad as NYC.”

- “Typically, it is fairly clear how to prioritize work vs family. Usually, family comes first except for time-limited and usually predictable circumstances at work, such as crunch-time for grants. Currently, they both need me … But largely I have had to tell my family to hold on, hopefully I'll be back in a couple of months. I am just hoping that modeling walking into the fight will benefit my sons.”

While most respondents were committed to their professional responsibilities, a few expressed personal fears associated with contact with COVID-19 patients or frustration with colleagues reticent to serve. One participant noted a pattern of inequity in which female colleagues were struggling with productivity, while male colleagues were accelerating in their careers.

### Enhanced Relationships With Colleagues

Respondents reflected on the emergence of community spirit, decreased competitiveness, increased collaboration and solidarity, and an increased sense of shared purpose in their professional workplace. Several respondents noted that a cohesive response to the pandemic had sparked a sense of inclusion, with enhanced relationships and pride at work (Table 2).

- “People in my community have brushed aside competitiveness and local politics to respond and work together.”

### Reassertion of Career Choice

A dominant theme in the response data from both physician investigators and PhDs was how the pandemic resulted in them reasserting heartfelt commitment to their choice of career, although 1 respondent considered leaving academia. Reaffirmation of career choice was largely due to realizing enhanced definition of the purpose of their work activities (Table 2).

- “Years into my practice, I was skeptical about my decision to become a physician. This was based on the administrative and regulatory burdens imposed by health insurance and hospital administrators. Since COVID-19, I rediscovered the higher purpose and passion that got me into health care to begin with, meaning the ability to help individuals through applied science and compassionate care.”

### Research

Both men and women, physicians and PhDs described concerns about their interrupted or delayed research or the required closure of their laboratories due to suspension of non–COVID-19 research. Many faculty viewed this negatively, although for some, the pandemic created opportunities (Table 3).

- “For those of us typically engaged in research outside of the infectious disease realm, we have had to halt much of our research as ‘nonessential,’ which has challenged the value that we typically put on those activities. In that respect, this crisis has prompted a recalibration in which more meaning is placed on patient-care and our community of caregivers and less on our own individual academic pursuits.”

The challenges included not being able to fulfill grant expectations and an adverse timing effect on laboratory animals that disrupted some experiments. Some feared that the setback would be permanent and that it affected their grants as well as their personal finances and own health. With experiments suspended, participants noted more time to complete data analysis and writing.

- “We have stopped all experiments, and the laboratory is focused on publishing manuscripts and writing/submitting grant applications.”

Nevertheless, others commented favorably on the opportunity presented by COVID-19 to reorient their research to improve the lives of individuals during these difficult times. The pandemic also highlighted burdensome competition for funding, and a very few considered reorienting their career away from research or academia.

- “This had made me rethink whether I want to get back on the hamster wheel of academia when this public health crisis is over.”

### Clinical Work

For some physicians, COVID-19 increased clinical volume, with more patients to see or fewer available team members to share the responsibilities, citing the redeployment of trainees to frontline work. As described earlier, COVID-19 also increased the sense of meaningfulness in clinical work, particularly for those on the frontlines (Table 3).

- “I have shifted to spending more time in the clinical arena with the COVID-19 outbreak, and I am quite content with my current workload mix.”

Some noted the stress associated with treating patients with COVID-19. Other faculty noted a shift toward telemedicine or decreased clinical work. For some, this came as a relief, while others regretted the inability to contribute to frontline needs.

### Health Disparities, Social Justice, and Advocacy

Respondents, including but not limited to URMM faculty, perceived that the adverse effects of COVID-19 were more marked for people of color. They described health disparities and fewer resources available to these communities. Faculty wanted to address these needs to reduce disparities.

- “[H]ow can I be a leader and help get a useful message out to the Latinx community to help them protect themselves and get the care they need?”

Several respondents channeled their efforts during the pandemic into advocacy. They felt motivated to advocate for those they identified as having more risk during the crisis, such as pregnant trainees as well as underserved patient populations. Some respondents described working internationally to assist with the COVID-19 crisis (Table 3).

### Family Responsibilities

A struggle for both physician investigators and PhD scientists was how to manage family responsibilities, and perceived conflict between their professional and family roles (Table 3). For parents with children at home, the stay-at-home orders and school closures demanded increased family focus. Many perceived this as compromising professional productivity and performance.

- “While I typically view things as a ‘see saw’ (rather than ’balancing’), where I focus largely on home issues then pivot to work issues; the pendulum is very heavily weighted towards home right now. Thus, I’m tested in not being able to spend as much time on work as I’d like (thus, compromising my desire to always do my best at work, but not feeling that way right now).”

Participants expressed concern about the risks of exposing family members to COVID-19. The pandemic prompted some respondents to reflect on what matters most as a family. Some contemplated their own mortality.

### Psychological Stress

Respondents reported feelings of disconnection and stress during the pandemic. For some respondents, well-being took on a new prominence. Sources of stress included increased demands of both work and family, disrupted research, and being engaged in work deemed nonessential, as opposed to direct efforts to fight the pandemic (Table 3).

- “[T]here are challenges regarding institutional policies that I am struggling to deal with … I am finding that the clinical work I am involved in can be more stressful. Also, my colleagues are equally stressed which makes the communications more challenging.”
- “At first, I felt disconnected that so many people were mobilized to do work that was relevant and I felt sidelined.”

### Leadership

The COVID-19 crisis caused respondents to reflect on leadership in their institutions. Several respondents greatly appreciated transparent communication from their institution during the crisis, particularly given limitations to personal protective equipment and reduced finances.

- “I have appreciated the leadership at my institution for their communications and adaptability to the circumstances. Even though we are sharing in the burden of reduced income across the hospital with temporary salary cuts, I have appreciated their honesty and transparency.”

Some faculty voiced concerns about deficiencies they perceived in leadership (Table 3). Faculty commented on the dearth of support provided by leaders for good scientists, and the slow implementation of the institutional response to requirements for patient care and providing personal protective equipment.

## Discussion

Despite recent accounts of negative mental health outcomes among health care professionals associated with the COVID-19 pandemic,^27,28,29,30,31,32,33^ our study adds to this literature by documenting physician investigators’ and PhD scientists’ perceptions of important positive impacts related to their experience of continuing to work during the pandemic in the US. Mental health findings notwithstanding, the huge challenges provided by COVID-19 seem to have additionally positively affected the vitality of our respondent pool of research faculty in medical schools and teaching hospitals. Descriptions of rediscovery of meaningfulness in work, whether in clinical care or in research, and the focus on applied science and compassionate care for all are a moving testament to the altruism and professional dedication of diverse groups of faculty in academic medicine. While a commitment to professionalism was widely expressed by respondents, commentary on gender inequity did surface, reflecting published evidence of the disproportionate adverse effect of the pandemic on women faculty.^34,35,36,37^

Our findings align with and build on prior research on faculty vitality^5^ in that the COVID-19 pandemic seemed to foster positive collegial relationships and community spirit; augmented the alignment of faculty core values with the work that faculty undertook; and provoked an enhanced awareness of meaningfulness of work, a sense of calling to medicine, gratitude, and renewed commitment to their careers and professional engagement. We note that our sample of faculty perceived heightened task significance and task identity due to COVID-19, contributing to meaningfulness of work^38^ and felt particularly affirmed by intrinsic motivators,^39,40^ aligned with self-determination theory.^41^ One novel external reward schema that emerged in COVID-19 was the concept of essential vs nonessential work. We hypothesize that faculty intrinsic motivators with associated vitality and values alignment were protective in the midst of crisis, warranting further study.

The COVID-19 pandemic demonstrated that physician investigators and PhD scientists are critical to the nation. However, numerous leaders have warned about the decreasing number of physician investigators nationally^42,43^ as well as the high institutional cost of faculty turnover. The retention rate of first-time NIH RO1 awardees in NIH funding averages only approximately 60% five years later. In this milieu, the reassertion of career commitment, increased vitality and human flourishing expressed by both physician investigators and PhDs, should be welcome news. We hope that the observed findings represent durable positive trends among faculty and help counteract the epidemic of burnout^4,44^ previously documented among faculty in academic health centers.

### Limitations

This study has limitations, including the inability to parse the perceived impacts of the pandemic in terms of chronology or region of the country. While location and intensity of COVID-19 varied across the US at the time of data collection, which was very early in the pandemic, the cohesive themes suggest that sources of vitality and challenges of the pandemic were shared by faculty across the nation. Due to the competitive research award prerequisite to participate in the institute, our sample does not represent all faculty or even all research faculty in medical schools. We do not know why these faculty applied to the C-Change Mentoring and Leadership Institute or whether their vitality differed from other research faculty who did not apply. However, we offered places to a random selection of physician and PhD applicants, stratified by gender and race/ethnicity, who met the inclusion criteria, and almost all invited applicants enrolled.

In addition, it was not possible to separate the perceived impact of the pandemic from current sociopolitical movements, including increased societal awareness of systemic racism, particularly anti-Black racism, which may also have affected participants’ focus on health disparities and social justice. As with any phenomenological research, we recognize that data were filtered through the lenses of the qualitative researchers. However, triangulating the coding of 3 interdisciplinary coders incorporated multiple perspectives to identify dominant themes.^25^ More expansive follow-up studies will help define the evolution of the perceived impact of COVID-19 on medical school faculty.

## Conclusions

In this qualitative study, diverse PhD and physician investigators reported experiencing increased meaningfulness of work and enhanced relationships with colleagues during the COVID-19 pandemic. These intrinsic motivators are associated with vitality. However, respondents also expressed increased mental health challenges. The findings of this study have implications for addressing burnout and faculty turnover. Institutions may wish to consider implementing practices to foster relational practices and intrinsic motivators^44,45,46,47^ in their faculty members to support faculty during times of high stress and to increase vitality, resilience, and ethical behaviors.
